# “Home is where the patient is”: a qualitative analysis of a patient-centred model of care for multi-drug resistant tuberculosis

**DOI:** 10.1186/1472-6963-14-81

**Published:** 2014-02-21

**Authors:** Shona Horter, Beverley Stringer, Lucy Reynolds, Muhammad Shoaib, Samuel Kasozi, Esther C Casas, Meggy Verputten, Philipp du Cros

**Affiliations:** 1 The London School of Hygiene and Tropical Medicine, London, UK; 2 Médecins Sans Frontières (MSF), London, UK; 3 MSF, Amsterdam, Netherlands; 4 The National TB and Leprosy Programme, Ministry of Health, Kampala, Uganda

**Keywords:** Multi-drug resistant tuberculosis (MDR-TB), Home-based care, Community, Ambulatory, Uganda, Acceptability, Qualitative, Transmission, Nosocomial

## Abstract

**Background:**

Ambulatory, community-based care for multi-drug resistant tuberculosis (MDR-TB) has been found to be effective in multiple settings with high cure rates. However, little is known about patient preferences around models of MDR-TB care. Médecins Sans Frontières (MSF) has delivered home-based MDR-TB treatment in the rural Kitgum and Lamwo districts of northern Uganda since 2009 in collaboration with the Ministry of Health and the National TB and Leprosy Programme. We conducted a qualitative study examining the experience of patients and key stakeholders of home-based MDR-TB treatment.

**Methods:**

We used semi-structured interviews and focus-group discussions to examine patients’ perceptions, views and experiences of home-based treatment and care for MDR-TB versus their perceptions of care in hospital. We identified how these perceptions interacted with those of their families and other stakeholders involved with TB. Participants were selected purposively following a stakeholder analysis. Sample size was determined by data saturation being reached within each identified homogenous category of respondents: health-care receiving, health-care providing and key informant. Iterative data collection and analysis enabled adaptation of topic guides and testing of emerging themes. The grounded theory method of analysis was applied, with data, codes and categories being continually compared and refined.

**Results:**

Several key themes emerged: the perceived preference and acceptability of home-based treatment and care as a model of MDR-TB treatment by patients, family, community members and health-care workers; the fear of transmission of other infections within hospital settings; and the identification of MDR-TB developing through poor adherence to and inadequate treatment regimens for DS-TB.

**Conclusions:**

Home-based treatment and care was acceptable to patients, families, communities and health-care workers and was seen as preferable to hospital-based care by most respondents. Home-based care was perceived as safe, conducive to recovery, facilitating psychosocial support and allowing more free time and earning potential for patients and caretakers. These findings could contribute to development of an adaptation of treatment approach strategy at national level.

## Background

Multi-drug resistant tuberculosis (MDR-TB) is defined as disease caused by *Mycobacterium tuberculosis* that is resistant to at least rifampicin and isoniazid. It can be acquired primarily through direct infection or can develop secondarily through inadequate treatment of drug-susceptible TB (DS-TB). Its treatment is lengthy, complex and can cause severe side effects. MDR-TB is an emerging issue globally; in 2011, 630 000 of the 12 million prevalent TB cases were MDR [1].

Historically, the model of MDR-TB treatment has been hospital-based, with patients admitted to a central institution for the intensive phase of treatment or longer, which usually means a minimum of 8 months [2]. Hospital-based models of treatment are costly and often unfeasible both for patients and health systems [3,4]. Only 16% of the cases of MDR-TB estimated to exist among notified TB patients were enrolled on treatment in 2010 [5].

Ambulatory, community-based care, whereby treatment is administered on an outpatient basis from a local community-based clinic often directly to patients in their homes (home-based care), has been found to be effective in multiple settings with high cure rates [4,6,7]. Treatment at the community level is feasible and safe and enables wider health influencing factors to be addressed including psychosocial support [8,9]. In addition, community-based models of care are three to four times cheaper than hospital-based approaches [3,10]. WHO now recommends MDR-TB treatment models that are primarily ambulatory [11,12]. However, little is known about patient and staff acceptability of community-based care in sub-Saharan Africa [13,14].

Uganda is classed as having a high burden of TB with approximately 63 000 TB cases in 2011 (183/100 000 population) [1]. An estimated 1.4% of new and 12% of previously treated TB cases are MDR, which means there were around 1000 new cases of MDR TB in 2011 [1]. Nearly 90% of Uganda’s population live in rural areas [15] and geographic access to health facilities is sub-optimal. 72% of people were reported as living within 5km of the nearest health facility in 2005, with the northern and eastern regions of Uganda having the poorest access to health-care services [16]. Kitgum and Lamwo are two adjacent rural districts in northern Uganda which are currently comparatively stable but historically have been subjected to conflict and displacement, with devastating effects on the socio-economic status of the population and health infrastructure.

Until recently there was no national MDR-TB treatment programme in Uganda, with Médecins Sans Frontières (MSF) being the sole treatment provider, delivering home-based MDR-TB treatment in Kitgum and Lamwo districts since 2009 in collaboration with the Ministry of Health (MoH) and the National TB and Leprosy Programme. The national DS-TB treatment provision has had associated drug shortages due to reasons including staff capacity at local and district level to prepare quality and timely anti-TB drug orders and at central level due to late preparation of drug orders and poor logistical support.

MDR-TB patients, under MSF’s home-based treatment and care programme, receive individualised treatment regimens according to drug-susceptibility testing (DST) results and WHO guidelines, following the DOT approach as part of the Stop TB Strategy, as well as receiving education, psychosocial support and enablers such as food rations. HIV co-infected patients in need of treatment are offered integrated HIV care within MDR-TB related activities. Initial treatment is delivered in a local isolation unit (at Madi Opei Health Centre, Lamwo district; or Kitgum Government Hospital) until the patient is clinically stable and can be accommodated in a separate sleeping hut (‘*tukul*’) at home. The remainder of the 18-24 months of treatment is then delivered at home six days per week, directly observed by health-care workers, who are employed by MSF.

The MSF MDR-TB programme has achieved good results [17]. However, the acceptability and accessibility of this model of care was not known. Although the Stop TB Strategy highlights the importance of patient-centred models of care [18] research on patients’ opinions and preferences is scarce. We conducted a qualitative study examining the experience of patients and key stakeholders with the aim of determining the acceptability and accessibility of the MDR-TB home-based treatment and care programmes in Kitgum and Lamwo. We also aimed to learn lessons from the programme implementation and obtain information to guide the development and future implementation of this model of care.

## Methods

We conducted a qualitative research study using semi-structured interviews and focus-group discussions in August and September 2011. We examined patients’ opinions, views and experiences of home-based treatment and care for MDR-TB versus their assertions of care in hospital. We also identified how these views compared with those of their families and other stakeholders involved with TB. These methods were chosen due to the exploratory nature of the research question, with interviews allowing an in-depth view to the opinions and experiences of respondents, and focus-group discussions showing group dynamics and general consensus versus anomalies regarding this topic. The principal investigator did not occupy the dual role of clinician and researcher, and therefore maintained a level of independence to reduce study bias.

Participants were identified and selected purposively following a stakeholder analysis, whereby all actors with a relationship to the topic (home-based treatment and care or MDR-TB) were mapped, and their likely view towards home-based treatment was rated from 1 to 5, with 1 being strongly negative view and 5 being strongly positive view. This mapping exercise also considered their likely influence, interest in the issue and any assumptions made. A range of different actors were then invited for interview through routine programme activities in order to explore the diversity of opinions on this topic. The principal investigator approached all identified participants outlining the study and requesting voluntary interview/focus-group discussion participation. Interviews and focus group discussions were audio-recorded and lasted around 60 and 90 minutes, respectively. Sample size was determined by data saturation being reached within each identified homogenous category of respondents: health-care receiving, health-care providing and key informants [19].

Interviews were based on the use of interview guides with prompts, allowing a participant-led and flexible approach to data collection. Interviews were structured around a core set of topics, including introductions, patient or family members’ experience of MDR-TB, as well as diagnosis and treatment; and their views on recommendations for future treatment provision. Conversation around these topics was participant-led and naturally flowed. Prompts and probes allowed particular topics to be explored in more depth, for example on asking patients if they spoke about having MDR-TB openly, it was then possible to explore their response to ascertain whether there was a fear of negative response or stigma. This approach allows for exploration of particular topics whilst still being participant-led and not risking asking leading questions. Topics used in health care provider and key informant interviews were slightly different, including an examination of the current system of treatment and care, acceptability of home-based care, adherence and stigma. An interpreter was used to translate questions and responses from Luo to English and vice versa in several interviews and discussions. A separate independent interpreter was used to re-translate interview recordings where possible to ensure validity of interpretations. Data were analysed throughout the entire course of the research, in that from the moment data were being generated the “thinking and theorising” began [20]. This iterative process of data collection and analysis enabled adaptation of topic guides and testing of emerging themes. For example, following analysis of initial patient interviews a code around patient adherence to MDR-TB treatment emerged, which led to this being added as a specific topic within interview guides in order for this to be explored further. Data were managed initially through verbatim transcription of all recorded conversational interviews. Systematic analysis of transcripts was conducted to identify codes, relevant themes, patterns and concepts compatible to a deductive grounded theory approach. A framework analysis was used to subdivide the data as well as assign categories to ascertain the most significant themes and patterns, with relationships between constructs, as well as deviant cases, being identified [20,21].

Formal ethics approval was gained from the London School of Hygiene and Tropical Medicine, MSF Ethics Review Board and Ugandan Ethics Review Board prior to the study commencing. Prior to interview commencement and recording all participants voluntarily gave their informed written consent. Confidentiality of respondents was ensured through the use of pseudonyms and data storage protection procedures. Feedback mechanisms were used to ensure participants were aware of the findings of the study and could choose to opt out.

## Results

### Participants

There were nine MDR and two mono-resistant patients receiving treatment at the time of the study; previously one MDR and three mono-resistant patients had completed treatment and been cured. All patients live in small villages in remote rural settings and 63% of the population in this region live below the poverty line [16].

Data saturation was reached in each respondent category after 30 interviews: 12 health-care receiving, nine health-care providing and nine key informant. Saturation was defined as occurring when themes that emerged in each respondent group were reiterated by different participants; and when additional participant transcript analysis revealed no new themes. Respondents included MDR-TB patients currently in the MSF home-based treatment and care programme (n = 7), their family members (5), health-care workers (6), village health team members (3) and identified key informants from the MoH (2) and non-governmental organisations (7). The two MDR-TB patients who did not participate in the study did so due to logistical reasons regarding time availability and working hours. All other approached participants voluntarily agreed to being interviewed.

Patients who agreed to participate were both male (4) and female (3), their ages ranged from 15 to 70 years and they were all from low socio-economic backgrounds, the majority being illiterate. Responses were analysed to account for gender and age differences; but these demographic factors did not influence the themes that emerged. A total of four focus group discussions were conducted: one with village health team members (6) and three with community members (10, 11 and nine participants). Village health team members are governmentally employed but in principle are volunteers, paid mainly in incentives and a small stipend to perform a role similar to community health workers. This focus group included three males and three females. The community member focus groups were held with members of a similar age and the same gender to minimise the effects of power dynamics. Two focus groups were held with groups of females (one with young females and one with older) and one was held with young men.

### Analysis

Several key themes emerged in relation to MDR-TB treatment: the asserted preference for and acceptability of home-based treatment and care as a model of MDR-TB treatment by patients, family, community members and health-care workers; the fear of nosocomial transmission of other infections within hospital settings; and the identification of MDR-TB developing through inadequate treatment regimens for DS-TB.

1. Perceived preference for home-based treatment and care and high acceptability

*‘I feel that getting treatment at home is better than hospital.’* Patient 01

All twelve patients and family members interviewed stated a preference for home-based care over hospital-based care, with some mentioning that this was conditional to the drug supply being constant and reliable. Acceptability of this model of care was high: home-based treatment and care was accepted by all health-care receiving respondents; the two district MoH respondents; all three village health team respondents; and five of the six health-care worker respondents. The one health-care worker respondent who was ambivalent was a nurse who feared secondary transmission within the community. The majority of community members who participated in focus group discussions stated a preference for home-based treatment and none mentioned a lack of acceptance of having patients in their communities being treated for MDR-TB at home. All members of village health teams interviewed said they would be willing to deliver MDR-TB treatment at home.

Home was perceived as an environment which:

•Is more conducive to recovery than hospital:

*‘I saw during the home-based care period patients get a lot of improvement in home than in the hospital.’* Family member 05

*‘I think from home someone recovers much faster than when hospitalised.’* Health-care worker 03

•Enables more psychosocial support due to the closeness of family and friends and perceived connectedness in comparison with feelings of isolation and loneliness associated with hospital admission:

*‘Having people coming home, chatting with you, it is nice and encourages you to take the drugs.’* Patient 02

•Provides free time for both the caretaker and the patient to conduct other activities such as performing small jobs and having social interactions.

Respondents also mentioned socioeconomic barriers to accessing treatment in hospital, including distance, affordability, transport costs, living costs while in hospital and distance from home creating indirect costs through lack of ability for the patient or caretaker to work. These barriers were seen as being particularly prohibitive of treatment access if the hospital were to be a centralised institution such as Mulago hospital in Kampala (the proposed provisory institute for treatment nationally), with the socio-economic factors mentioned above as well as language barriers making the vast majority of patients and family members feel they would be unable to receive treatment from this location.

*‘If I was in the hospital in Kampala the cost for transport is very expensive and someone must come to see you and stay for 1 month maybe, then they cannot do other things in this time and it would be very costly.’* Patient 02

*‘Because the patient comes from a poor family, he cannot raise the money to go to Mulago* [National Referral Hospital in Kampala]*’* MoH 02

Home-based treatment and care was therefore seen to be more accessible to patients and their family members, as well as more acceptable to both health-care receiving and health-care providing respondents.

2. Fears of transmission of MDR-TB and other infections

No patient or family respondents mentioned fears or association of treatment at home with heightened risk of secondary transmission of MDR-TB. However, three health-care workers and several key informant respondents mentioned fears of potential MDR-TB transmission with home-based care and highlighted the need for isolation of patients during the initial phase of treatment, either in a special *tukul* at home or in a local isolation ward. Respondents mentioned perceiving hospital as being an environment that poses greater risk for catching other infections via nosocomial transmission and the majority of respondents felt the home environment would be more protective of patients’ health in this regard.

Community members in general were positive about the idea of patients being treated at home: the majority of women and young women in two focus groups thought home was the best place for people with MDR-TB to be treated; and nearly half the young men in another focus group preferred home - the other half were either unsure or said they would prefer hospital due to perceptions that treatment would be more likely to be delivered on time. No participants of these community focus group discussions mentioned a fear of MDR-TB transmission to themselves associated with patients being treated at home within the community.

Several respondents mentioned the existence of stigma towards MDR-TB patients, in each case linked to the association of MDR-TB with HIV. The stigma perceived or enacted was not due to the MDR-TB itself but rather its association with HIV, and was said to usually come in the form of rumours. Participants felt that this was due to health promotion messages that state that HIV and TB go hand in hand and therefore someone with TB is assumed to be HIV positive. In addition, symptoms were perceived as being similar for both conditions.

*‘Actually because when you see the signs and symptoms of HIV compared with that of tuberculosis, normally they are similar… loss of weight, the appearance of the face, the hair, moves similar to that of HIV.’* Focus group discussion 04 – young males

The vast majority of respondents who observed stigma either felt or had seen this being overcome by community sensitisation:

*‘After the sensitisation within here there is now nothing, people are living normal lives, they are supporting us and others with ideas, we can chat freely.’* Family member 01

3. Inadequate treatment of DS-TB

Nine of the 12 health-care receiving respondents felt they could identify the origin of patients’ MDR-TB. Of these nine, most associated MDR-TB development with interruptions in nationally provided DS-TB treatment. Reasons cited included a lack of drug availability (with patients mentioning going to health clinics for drug refills and finding there were no drugs), lack of staff availability for dispensing refills and procurement of expired drugs:

*‘I used to go to the hospital and there would be no drugs’* Patient 01

*‘I was taking expired drugs for a very good period’* Patient 06

Several health-care workers echoed these views on treatment availability for DS-TB, mentioning lack of consistency in drug supplies, low drug stocks and periods when there were no drugs available.

Some health-care workers and key informants associated MDR development with the adherence of DS-TB patients as well as treatment shortages:

‘*It is an issue of adherence, they stop start stop start if they feel better.’* Key informant 02

Respondents’ opinions on MDR-TB development in this context highlight potential issues in need of attention, including patient education to improve adherence to DS-TB treatment and improvements in drug supply.

## Discussion

The results show that home-based treatment and care was acceptable to patients, families, communities and health-care workers and was seen as preferable to hospital-based care by most respondents in all groups, including by all interviewed patients and family members. Home-based care was also viewed as safe, conducive to recovery, facilitating psychosocial support and allowing more free time and earning potential for patients and caretakers. While some health-care workers were concerned about infection transmission risks with home-based care, patients were instead concerned about hospital-acquired infections. In addition to MDR-TB treatment, MSF provided information on MDR-TB and infection control to the patient and their extended household, including education around modes of transmission. Patients were isolated in a separate Tukul (hut) within the home during the initial phase of treatment, while they were sputum positive, the logistics of which were supported by MSF. Home visits were conducted by MSF medical staff and a counsellor, who paid attention to the patient’s experience as well as perceptions of the family. These factors would have influenced the results with regards to assertions around MDR-TB transmission.

Most participants identified MDR-TB as having developed from DS-TB treatment interruptions. That certain health-care workers and key informants also associated MDR development with DS-TB patient adherence is a crucial point which warrants further investigation into the treatment for DS-TB and specific action targeting adherence to this treatment.

The need for a treatment strategy in Uganda, which rapidly and adequately treats MDR-TB and HIV has been highlighted elsewhere, with one study finding the retreatment approach to TB to be unsatisfactory [22]. Our study is the first to assess the acceptability of a model of home-based treatment and care for MDR-TB for patients and staff. However, this is complemented by several studies examining community-based models of treatment for DS-TB and MDR-TB in multiple settings, including Uganda. These show community or home-based treatment to be effective, feasible and safe [6-8] as well as supportive of adherence [23].

Qualitative research exploring patients’ responses to various models of MDR-TB treatment is lacking, and thus our study provides an insight into a patient-centred approach to assessing this model of treatment and care. In qualitative research examining the DOTS approach for DS-TB, support and treatment delivery that were patient-centred, rather than enforced via inspectorial observation, resulted in higher treatment uptake and adherence [24] as did the addition of social and psychological support [23]. Other research has highlighted the need for treatment that goes beyond provision of anti-TB drugs to include care and support delivered via ground-up models [25] and a focus on patient contacts as well as patients themselves [26]. These findings are in line with Component 5 of the Stop TB Strategy, outlining the need for patient and community involvement in TB care to achieve sustainable and effective TB initiatives [18].

Health and treatment outcomes have been shown to be influenced by the psychosocial and economic aspects of treatment and care for MDR-TB [9]. In our study, respondents described factors relating to health and treatment outcomes as being enhanced through home-based care. Socioeconomic access barriers were mentioned in association with hospital-based but not home-based care. A similar finding was noted in qualitative research examining the DOTS approach for DS-TB, with barriers described including patients not being able to undertake treatment due to cost of transport to services and loss of earnings through inability to work [24]. In our study, respondents noted that home-based care allowed psychological support from family members and social contact with friends and community members. They also described close relationships with MSF staff including counsellors and the health-care workers who directly observed treatment. These factors were seen to facilitate the treatment process, giving strength of mind and positivity to patients and encouraging them with adherence. Respondents did not mention any patients experiencing difficulties with adherence, however this topic was not examined specifically and thus conclusions cannot be drawn. All MDR-TB patients interviewed mentioned the importance of being able to have free time for other activities.

Our study showed that some health-care workers were concerned about the risk of transmission of MDR-TB if patients were treated at home, while conversely patients were concerned about the risk of catching other infections in hospital. The perceptions of the patients reflect findings that there are fewer opportunities for transmission of other infections with home-based MDR-TB treatment and care [4]. Further research is required to examine secondary transmission of MDR-TB in both hospital and community treatment models. Our results demonstrated that fears of MDR-TB infections lay with health-care professionals rather than with health-care receivers, which warrants further investigation into how such fears might guide health-care worker practice and whether these fears are warranted. This finding also calls for programmatic attention to ensure adequate training of staff on the risks of nosocomial transmission and transmission prevention measures used in home-based treatment settings.

Respondents outlined the existence of weaknesses within current national DS-TB health services including the provision of expired drugs, lack of drug availability and drug stock outs, lack of staff availability for refill dispensing, and lack of support and information to encourage patients to adhere to treatment. There is need for adequate treatment provision for DS-TB in order to prevent the selection of naturally occurring mutations that cause MDR-TB to develop [27]. Our results highlighted the importance of a strengthened drug supply line to villages, since the role of supply interruptions for TB medication in development of MDR resistance was acknowledged by both patients and medical staff. The negative effects of drug stock outs have been shown in HIV, though there is a dearth of research in this area for TB [28]. To address the issue of interrupted drug supply lines, MSF has trained suppliers and relevant Ministry of Health staff in medical supply ordering processes.

In July 2012, Uganda’s Ministry of Health began rolling out its first national treatment programme for MDR-TB, which aims to treat around 250 already confirmed cases. The proposed treatment strategy involves initial hospitalisation followed by ambulatory care with patients being monitored by regional health centres and treatment delivered at the community level by village health teams. Our findings on patients’ experiences of home-based care could be used to contribute to adaptation of treatment approach strategy at national level.

### Limitations

Our study design was explorative and the findings are not generaliseable to all contexts and settings. However, our work highlights issues likely to be relevant to many, particularly those with a similarly high proportion of rurally situated patients with low socioeconomic status; and is supported by findings that ambulatory or community-based care is feasible and effective. Analysis of participant accounts is interpretative and involves the “positioned” researcher [29]. Reflexivity about the influence of the researcher on shaping the data was ensured through awareness of potential researcher biases and analysing verbatim transcriptions word-for-word to avoid interpretation of meaning by the researcher. Coding and emergent themes were also checked by a second researcher to minimise potential bias. The principal investigator is a young white British female; the power dynamic of researcher and participant has been acknowledged throughout data collection and analysis and has been minimised by the fact that the principal investigator had no responsibility for programme management or patient care and was an outsider to routine programme activities.

The design of this study precluded collection of data on resistance patterns and increase in vulnerability to the development of resistance. While it demonstrates that fears of barriers to treatment created by stigma and community rejection are unfounded, this present proof of acceptability to patients and communities is not in itself adequate to demonstrate that home-based care for MDR-TB is equally effective or superior to hospital-based care as we have not assessed the effect of the extension of current supply lines for MDR-TB medications that would be needed in an expanded home-based care model.

## Conclusion

In a rural setting in Uganda, home-based treatment and care for MDR-TB was found to be preferred by patients and staff to hospital-based care for three main reasons: it was seen as being more conducive to patient recovery, it enabled enhanced psychosocial support and it allowed more free time for patients and caretakers to conduct other activities. Patients and staff viewed home-based treatment and care to be patient-centred, accessible, acceptable and feasible in this setting. Assertions on nosocomial transmission and drug supply interruptions highlight the need for improved training on infection control for health-care workers.

It is essential not to overlook the importance of reliable drug supply lines and buffer stock management for DS and MDR-TB programmes, with contingency planning in case of failure of supply lines. Studies to research this issue are currently lacking but are urgently needed to inform programming decisions and donor choices.

### RATS guidelines

This study adheres to the RATS Guidelines on qualitative research.

## Competing interests

During the past five years, LR has been employed as an MSF Field Coordinator and a Head of Mission. She has no other relevant interests. All other authors declare that they have no competing interests.

## Authors’ contributions

SH acted as the principal investigator; the study was conducted in fulfilment of the research project component of the MSc in Control of Infectious Diseases. She participated in the design of the study, carried out the acquisition of data by conducting interviews and focus group discussions, transcribed, analysed and interpreted data and findings, drafted and revised the manuscript. She is the guarantor of the study. BC was involved in study conception and design, supported analysis and reviewed drafts. LR set up the project team by offering MSF UK the opportunity of a research cooperation of the London School of Hygiene and Tropical Medicine, and identified SH as an experienced INGO consultant well suited to conducting a study which could meet the need identified in the MSF-Operational Centre Amsterdam project in Uganda for an evaluation of the acceptability and functioning of their home-based MDR treatment programme in the Kitgum region. She suggested the use of rapid rural appraisal techniques for this enquiry, provided training in these techniques in the academic context for SH as the principal investigator, co-supervised the design and content of the study with PD and BC, and finally provided feedback on the findings and their presentation through the marking procedure as SH’s Project Supervisor at LSHTM. MS facilitated in the implementation of the study and reviewed drafts. SK contributed to design, acquisition of data and was involved in the revision of the draft manuscripts. EC reviewed drafts. MV reviewed drafts. PdC conceived and participated in the design of the study, and reviewed drafts. All authors read and approved the final manuscript.

## Pre-publication history

The pre-publication history for this paper can be accessed here:

http://www.biomedcentral.com/1472-6963/14/81/prepub

## References

[B1] WHO Global Tuberculosis Report 2012 2012 Geneva: WHO

[B2] WHO Multidrug and Extensively Drug-Resistant TB (M/XDR-TB): 2010 Global Report on Surveillance and Response 2010 Geneva: WHO

[B3] Fitzpatrick C Floyd K A systematic review of the cost and cost effectiveness of treatment for multidrug-resistant tuberculosis Pharmacoeconomics 2012 14 63 80 10.2165/11595340-000000000-00000 22070215

[B4] Smart T Decentralised, patient-centred models of delivering treatment and palliative care for people with M/XDR-TB HATIP 2010 14 2 9 Available: https://www.aidsmap.com/pdf/HATIP-166-October-8th-2010/page/1520450/ Accessed Jan 28, 2011

[B5] WHO Global Tuberculosis Control 2011 2011 Geneva: WHO

[B6] Mitnick C Bayons J Palacios E Shin S Furin J Alcantara F Sanchez E Sarria M Becerra M Smith Fawzi M Kapiga S Neuberg D Maguire J Yong Kim J Farmer P Community-based therapy for multidrug-resistant tuberculosis in Lima, Peru NEJM 2003 14 119 128 10.1056/NEJMoa022928 12519922

[B7] Luyirika E Nsobya H Batamwita R Busingye P Musoke W Nabiddo L Karamagi Y Mukasa B A home-based approach to managing multi-drug resistant tuberculosis in Uganda: a case report AIDS Res Ther 2012 14 12 10.1186/1742-6405-9-12 22524486 PMC3349607

[B8] Heller T Lessells R Wallrauch C Barnighausen T Cooke G Mhlongo L Master I Newell M Community-based treatment for multidrug-resistant tuberculosis in rural KwaZulu-Natal, South Africa Int J Tuberc Lung Dis 2010 14 420 426 20202299

[B9] Chalco K Wu D Mestanza L Munoz M Llaro K Guerra D Palacios E Furin J Shin S Sapag R Nurses as providers of emotional support to patients with MDR-TB Int Nurs Rev 2006 14 253 260 10.1111/j.1466-7657.2006.00490.x 17083413

[B10] Nathanson E Lambregts-van Weezenbeek C Rich M Gupta R Bayona J Blondal K Caminero J Cegielski P Danilovits M Espinal M Hollo V Jaramillo E Leimane V Mitnick C Mukherjee J Nunn P Pasechnikov A Tupasi T Wells C Raviglione M Multidrug-resistant tuberculosis management in resource-limited settings Emerg Infect Dis 2006 14 1389 1397 10.3201/eid1209.051618 17073088 PMC3294733

[B11] WHO Treatment of Tuberculosis Guidelines 2010 4 Geneva: WHO 23741786

[B12] WHO Guidelines for the Programmatic Management of Drug-Resistant Tuberculosis: 2011 Update 2011 Geneva: WHO

[B13] Furin J Bayona J Becerra M Farmer P Golubkov A Hurtado R Joseph JK Keshavjee S Ponomarenko O Rich M Shin S Programmatic management of multidrug-resistant tuberculosis: models from three countries Int J Tuberc Lung Dise-publication ahead of print 8 June 2011. doi:10.5588/ijtld.10.0591 10.5588/ijtld.10.0591 21669029

[B14] Palacios E Guerra D Llaro K Chalco K Sapag R Furin J The role of the nurse in the community-based treatment of multidrug-resistant tuberculosis (MDR-TB) Int J Tuberc Lung Dis 2003 14 343 346 12729339

[B15] World Bank Uganda at a glance – World Bank Available: devdata.worldbank.org/AAG/uga_aag.pdf Accessed October 26, 2012

[B16] Kiwanuka SN Ekirapa EK Peterson S Okui O Hafizur Rahman M Peters D Pariyo GW Access to and utilisation of health services for the poor in Uganda: a systematic review of available evidence Trans R Soc Trop Med Hyg 2008 14 11 1067 1074 10.1016/j.trstmh.2008.04.023 18565559

[B17] Shoaib M Velivela K Sharmin S Seshadri P Kasozi S Casas EC Verputten M A decentralised, community-based model of care for DR-TB in northern Uganda. Poster presented at MSF Scientific Day 2012 Available: http://issuu.com/msfuk/docs/verputten_uganda_drtb/1 Accessed Oct 23, 2012

[B18] WHO The Stop TB Strategy: Building on and Enhancing DOTS to Meet the TB-Related Millennium Development Goals 2006 Geneva: WHO

[B19] Green J Thorogood N Qualitative Methods for Health Research 2009 London: Sage Publications

[B20] Basit T Manual or electronic? The role of coding in qualitative data analysis Educ Res 2003 14 143 154 10.1080/0013188032000133548

[B21] Bradley E Curry L Devers K Qualitative data analysis for health services research: developing taxonomy, themes and theory Health Serv Res 2007 14 1758 1772 10.1111/j.1475-6773.2006.00684.x 17286625 PMC1955280

[B22] Jones-López EC Ayakaka I Levin J Reilly N Mumbowa F Dryden-Peterson S Nyakoojo G Fennelly K Temple B Nakubulwa S Joloba ML Okwera A Eisenach KD McNerney R Elliott AM Ellner JJ Smith PG Mugerwa RD Effectiveness of the standard WHO recommended retreatment regimen (Category II) for tuberculosis in Kampala, Uganda: a prospective cohort study PLoS Med 2011 14 e1000427 10.1371/journal.pmed.1000427 21423586 PMC3058098

[B23] Gelmanova IY Taran DV Mishustin SP Golubkov AA Solovyova AV Keshavjee S ‘Sputnik’: a programmatic approach to improve tuberculosis treatment adherence and outcome among defaulters Int J Tuberc Lung Dis 2011 14 1373 1379 10.5588/ijtld.10.0531 22283898

[B24] Noyes J Popay J Directly observed therapy and tuberculosis: how can a systematic review of qualitative research contribute to improving services? A qualitative meta-synthesis J Adv Nurs 2007 14 227 243 10.1111/j.1365-2648.2006.04092.x 17233644

[B25] Koch E Tuberculosis is a threshold: the making of a social disease in post-Soviet Georgia Med Anthropol 2013 14 309 324 10.1080/01459740.2012.751384 23768217

[B26] Cavalcante SC Durovni B Barnes GL Souza FB Silva RF Barroso PF Mohan CI Miller A Golub JE Chaisson RE Community-randomized trial of enhanced DOTS for tuberculosis control in Rio de Janeiro, Brazil Int J Tuberc Lung Dis 2010 14 203 209 20074412 PMC3812056

[B27] Temple B Ayakaka I Ogwang S Nabanjja H Kayes S Nakubulwa S Worodria W Levin J Joloba M Okwera A Eisenach KD McNerney R Elliott AM Smith PG Mugerwa RD Ellner JJ Jones-López EC Rate and amplification of drug resistance among previously-treated patients with tuberculosis in Kampala, Uganda Clin Infect Dis 2008 14 1126 1134 10.1086/592252 18808360 PMC2883442

[B28] Pasquet A Messou E Gabillard D Minga A Depoulosky A Deuffic-Burban D Losina E Freedberg KA Danel C Anglaret X Yazdanpanah Y Impact of drug stock-outs on death and retention to care among HIV-infected patients on combination antiretroviral therapy in Abidjan, Cote d’Ivoire PLoS One 2010 14 e13414 10.1371/journal.pone.0013414 20976211 PMC2955519

[B29] Malterud K Qualitative research: standards, challenges and guidelines Lancet 2001 14 483 488 10.1016/S0140-6736(01)05627-6 11513933

